# Mechanism of mitomycin-induced apoptosis in cultured corneal endothelial cells

**Published:** 2008-09-15

**Authors:** Kwou-Yeung Wu, Hwei-Zu Wang, Show-Jen Hong

**Affiliations:** 1 Department of Ophthalmology, Faculty of Medicine, College of Medicine, Kaohsiung Medical University, Kaohsiung, Taiwan; 2 Department of Ophthalmology, Kaohsiung Medical University Hospital, Kaohsiung, Taiwan; 3 Department of Pharmacology, Faculty of Medicine, College of Medicine, Kaohsiung Medical University, Kaohsiung, Taiwan

## Abstract

**Purpose:**

Previous studies have indicated that improper use of mitomycin C (MMC) caused cytotoxicity in corneal endothelial cells. The aim of the present study was to investigate whether MMC induces cellular apoptosis in corneal endothelial cells and to determine the mechanism by which this may occur.

**Methods:**

Porcine corneal endothelial cells were acquired from primary culture. Cellular damage and caspase pathway were estimated with a MTT (3-[4,5-dimethylthiazol-2-yl]-2,5-diphenyl tetrazolium bromide) assay. The apoptotic characteristics were detected by means of flow cytometry, the TUNEL (terminal deoxyribonucleotidyl-transferase-(TdT)-mediated deoxyuridine-5′-triphosphate-digoxigenin (dUTP) nick-end labeling) test, immunofluorescent staining, and western blotting.

**Results:**

The results indicated that MMC was toxic to corneal endothelial cells in a time-dependent and dose-dependent manner. Pretreatment with a general caspase inhibitor (Z-VAD-FMK), a caspase-8 inhibitor (Z-IETD-FMK), and a caspase-9 inhibitor (Z-LEHD-FMK) reversed MMC-induced cellular damage. Following exposure to MMC, a change in the mitochondrial membrane potential was positively detected by flow cytometric assay with MitoLight dye while cellular cytochrome *c* that was released from the mitochondria to the cytoplasm was detected by immunofluorescent staining. A positive TUNEL test revealed that cellular DNA apoptosis had occurred following exposure to 0.001 and 0.01 mg/ml MMC for 24 h. Positive annexin V-FITC, and negative propidium iodide (PI) staining indicated that the cellular plasma membrane underwent apoptosis following 0.001 mg/ml MMC exposure for 24 h. Western blot assay demonstrated down-regulation of the Bcl-2 protein and upregulation of the p53 and p21 proteins, which were all involved in apoptosis induced by MMC.

**Conclusions:**

These results indicate that mitomycin-induced cellular apoptosis in corneal endothelial cells may be mediated through caspase-8, caspase-9, and the mitochondrial regulated pathways as well as through upregulation of p53-dependent and p21-dependent signal transduction pathways.

## Introduction

Adjuvant use of mitomycin C (MMC) is prevalent in the treatment of various ocular diseases including pterygium [1], glaucoma [2], and refractive surgery [3] to improve the success of clinical therapy. MMC is applied by topical drops, local soaking, or subconjunctival injection during or after pterygium [4] and glaucoma [5] surgeries to inhibit cellular proliferation. For laser refractive surgery, MMC is applied with local soaking to the cornea to modulate corneal keratocyte growth and to decrease the postoperative corneal opacity [6]. When MMC is topically soaked onto the pterygium area, a scleral flap, or the cornea, a certain amount of MMC may penetrate into the corneal endothelium where it causes toxicity to corneal endothelial cells. In clinical observations, a significant loss of these cells is noted after trabeculectomy with adjunctive MMC soaking [7]. Evidence has also presented that a single application of MMC onto the corneal surface can cause dose-dependent corneal edema and endothelial apoptosis in a rabbit model system [8]. Thus, improper use of MMC as an ocular medication may result in damage to the cornea and impair the physiologic function of the corneal endothelium.

It is known that corneal endothelial cells play a crucial role in maintaining corneal transparency. Corneal clarity requires a net movement of fluid from the corneal stroma to the aqueous humor [9], and the efficiency of this flux depends on the presence of undamaged corneal endothelial cells and adequate cellular density. Any factor that decreases cell density in the endothelium may also reduce the efficiency of the corneal pump function and therefore affect transparency [10].

We have previously demonstrated that MMC is toxic to corneal endothelial cells in a time-dependent and dose-dependent manner [11]. Exposure of corneal endothelial cells to MMC for a certain dose and incubation time may induce apoptosis. This is a process of natural cell death that is characterized by an extreme heterogeneity of signal transduction pathways that lead to DNA degradation and dysfunctions of the plasma membrane and mitochondria, which are accompanied by a series of degenerative pathways [12,13]. To evaluate the potential chronic toxicity of MMC, cultured porcine corneal endothelial cells were used in the present study to investigate the apoptotic characteristics and mechanisms involved in corneal damage induced by MMC.

## Methods

### Materials

Cell culture materials including trypsin, minimal essential medium (MEM), glutamine, gentamicin, and fetal bovine serum were obtained from Gibco (Grand Island, NY). MTT (3-[4,5-dimethylthiazol-2-yl]-2,5-diphenyl tetrazolium bromide) was purchased from Sigma Chemical (St. Louis, MO). The general caspase inhibitor, Z-VAD-FMK; caspase-8 inhibitor, Z-IETD-FMK; caspase-9 inhibitor, Z-LEHD-FMK; and a TUNEL (terminal deoxyribonucleotidyl-transferase-(TdT)-mediated deoxyuridine-5′-triphosphate-digoxigenin (dUTP) nick-end labeling) apoptosis detection kit were purchased from Calbiochem (Bad Soden, Germany). A MitoLight mitochondrial apoptosis detection kit was purchased from Chemicon International Inc. (Temecula, CA). Rabbit antibodies to cytochrome *c* and p21 were purchased from Santa Cruz Biotechnology Inc. (Santa Cruz, CA). Alexa Fluor 488-conjugated goat anti–rabbit antibody; the annexin V– FITC/propidium iodide double staining kit; and mouse anti–human antibodies to Bcl-2 and p53 were purchased from Invitrogen (Carlsbad, CA). Western Blot Chemiluminescence Reagent Plus was purchased from New England Nuclear (PerkinElmer, Boston, MA). Protein assay dye and agents used for electrophoresis were purchased from Bio-Rad (Richmond, CA). Horseradish peroxidase-conjugated sheep anti-mouse IgG and donkey anti–rabbit IgG were obtained from Amersham Pharmacia (Buckinghamshire, England). Mitomycin C (MMC) was purchased from Kyowa (Hakko Kogyo Co., Tokyo, Japan). All other chemicals were obtained from Merck (Darmstadt, Germany).

### Culture of porcine corneal endothelial cells

Culture of porcine corneal endothelial cells was performed as published previously [11]. Briefly, porcine eyeballs were collected from a local slaughterhouse. Under sterile conditions, the corneal endothelial cells were separated using 0.25% trypsin for 30 min. The action of trypsin was stopped by adding minimal essential medium (MEM) containing 10% fetal bovine serum, 3.8 mM L-glutamine, and 50 μg/ml gentamicin. After centrifugation, the cells were resuspended in a culture flask. The culture was kept in a humidified chamber of 5% CO_2_ at 37 °C, and the medium was changed every two to three days.

### Assay of cell viability with MTT

Cell viability was measured with MTT dye following previously published procedures [14]. The MTT assay is based on the production of purple formazan from a methyl tetrazolium salt by the mitochondrial enzymes of viable cells. Cultured cells at a concentration of 4,000 cells/well were seeded in 96 well culture plates and allowed to form a monolayer for 24 h. The cells were then exposed to 150 μl of serum-free MEM medium containing various concentrations of MMC for 15 h and 24 h. For analysis of apoptotic caspase pathways, cells were pre-incubated with different caspase inhibitors including a general caspase inhibitor (Z-VAD-FMK), a caspase-8 inhibitor (Z-IETD-FMK), and a caspase-9 inhibitor (Z-LEHD-FMK) for 1 h before adding 0.001 mg/ml MMC. After exposure to the drug for 24 h, cells were washed twice with phosphate-buffered saline (PBS) and incubated with 150 μl MTT solution (0.833 mg/ml in PBS) for 4 h at 37 °C. At the end of the incubation period, the MTT solution was carefully aspirated, taking care not to disturb the crystal of purple formazan at the bottom of each well. The formazan reaction product was dissolved by the addition of 150 μl dimethyl sulfoxide (DMSO), and the optical density of the fluid in each well was read at 510 nm in a multi-well spectrophotometer (Titertek Multiscan, Flow Lab, Scotland, UK). Cytotoxicity was calculated based on a significant difference in the optical density between the MMC-treated and control groups.

### Immunofluorescent staining of cytochrome *c* protein in corneal endothelial cells

Coverslips coated with a confluent monolayer of cells were treated with MMC following previously published procedures [15]. Cells were washed twice with PBS and rinsed with 0 °C acetone for 20 s. Following a thorough PBS washing, rabbit anti-cytochrome *c* antibody (1:50) was applied to cells in a humidified chamber at 37 °C for 1 h. Cells were again washed with PBS and then incubated with Alexa Fluor 488-conjugated goat anti–rabbit antibody (1:200) for 1 h at room temperature. The stained samples were then rinsed and mounted. All slides were examined and photographed with a fluorescence microscope (Leitz Ortholuxix II; Leitz, Wetzlar, Germany).

### Flow cytometry assay of apoptotic changes in plasma membrane and mitochondrial membrane potential

Apoptosis in the plasma membrane was identified by flow cytometry with annexin V-FITC/propidium iodide (PI) staining. Mitochondrial membrane potential changes were assayed with MitoLight dye. In healthy cells, this dye accumulates in the mitochondria and yields a red fluorescence. In apoptotic cells where mitochondrial membrane potential has been depolarized, the dye aggregates in the cytoplasm and gives off a green fluorescence, allowing discrimination of apoptotic and non-apoptotic cells. In the absence or presence of various concentrations of MMC, cells were incubated with 1 μg/ml annexin V-FITC and PI for 10 min or with 50 μl of pre-diluted MitoLight solution (900 μl of water, 1 μl of MitoLight dye, and 100 μl of 10X  incubation buffer) for 15 min following the manufacturer’s instructions. Cells were then analyzed by flow cytometry (Becton, Dickinson Inc., Franklin Lakes, NJ) for a cell count of 10000.

### DNA apoptotic TUNEL staining

Immunohistochemical evidence for DNA strand breaks was obtained using the TUNEL assay. The cells were plated on coverslips for at least 24 h for attachment and then incubated with various concentrations of MMC at 37 °C. After exposure to MMC, the cells were washed twice with TBS (20 mM Tris-HCl, 140 mM NaCl, pH 7.6). According to the manufacturer’s instruction, the monolayer of cells was fixed for 20 min in 4% formaldehyde at room temperature and then washed three times in TBS. Cell membrane permeability was increased by treating the samples with 20 μg/ml proteinase K solution at room temperature for 5 min. Endogenous peroxidase activity was quenched by immersing the specimens in 2% H_2_O_2_ at room temperature for 5 min. The samples were rinsed by replacing the hydrogen peroxide solution with labeling buffer and reaction mixture solution. The specimens were then placed in a humidified incubator for 1 h at 37 °C. The labeling reaction was stopped by immersion in stop buffer at room temperature for 5 min. The specimens were subsequently washed in TBS. Then, 100 μl of streptavidin-horseradish peroxidase was applied to the cells for 20 min at room temperature. After washing with TBS, the specimens were immersed in diaminobenzidine (DAB) solution at room temperature for 5–10 min until a satisfactory color reaction was achieved. This assay was validated with the use of control slides, which had been ascertained to contain apoptotic (positive control) and non-apoptotic (negative control) cells.

### Western blot assay of proteins involved in apoptosis

Bcl-2, p53, and p21 proteins were detected by sodium dodecyl sulfate (SDS)–PAGE following a previously published procedure [15]. Briefly, cells were treated with MMC and washed with 10 ml of buffer A (20 mM of N-2-hydroxyethyl-1-piperazine-N'-2-ethanesulfonic acid [HEPES], 1 mM of ethylene diamine tetraacetic acid [EDTA], 2 μg/ml aprotinin, 2 μg/ml leupeptin, and 1 μg/ml pepstatin A, pH 7.4). Cells were then scraped into the ice-cold buffer A and immediately homogenized. Protein concentrations were determined with Bio-Rad protein assay dye. Protein (10 μg) from each sample was added to the SDS-PAGE sample buffer and heated in a boiling water bath for 5 min. An aliquot was then subjected to 10% SDS–PAGE. The proteins separated by SDS–PAGE were transferred in a Bio-Rad Trans-Blot cell onto nitrocellulose membranes. Transfer was performed at 100 V for 2 h in a buffer containing 25 mM Tris-HCl, 190 mM glycine, 0.01% SDS, and 20% methanol. The blots were blocked at room temperature for 1 h with buffer solution (20 mM Tris-HCI, 137 mM NaCl, pH 7.6) containing 5% nonfat milk and 0.1% Tween-20. Incubation was then performed for 1 h at 37 °C with monoclonal anti-Bcl-2 (1:50), anti-p53 (1:500), or rabbit anti-p21 (1:250) antibodies. The nitrocellulose membrane was washed three times with the same buffer used during the blocking phase and incubated with horseradish peroxidase-conjugated sheep anti-mouse IgG (1:2,000) or donkey anti–rabbit (1:10,000) as the secondary antibody at room temperature for 1 h. After washing, immunocomplexes were visualized by adding Western Blot Chemiluminescence Reagent Plus. The molecular size of the immunoreactive bands was determined by comparing them with a set of molecular weight marker proteins (Bio-Rad). Relative band intensity was then analyzed using the LabWorks software 4.6 from UVP Bioimaging systems (Upland, CA).

### Statistical analysis

Data were analyzed by one way ANOVA followed by Dunnett’s post hoc analysis. The values were expressed as mean±standard deviation (SD). All data were significantly different at p<0.05.

## Results

### Effects of mitomycin C on cell viability and apoptotic caspase pathways

**Figure 1:**
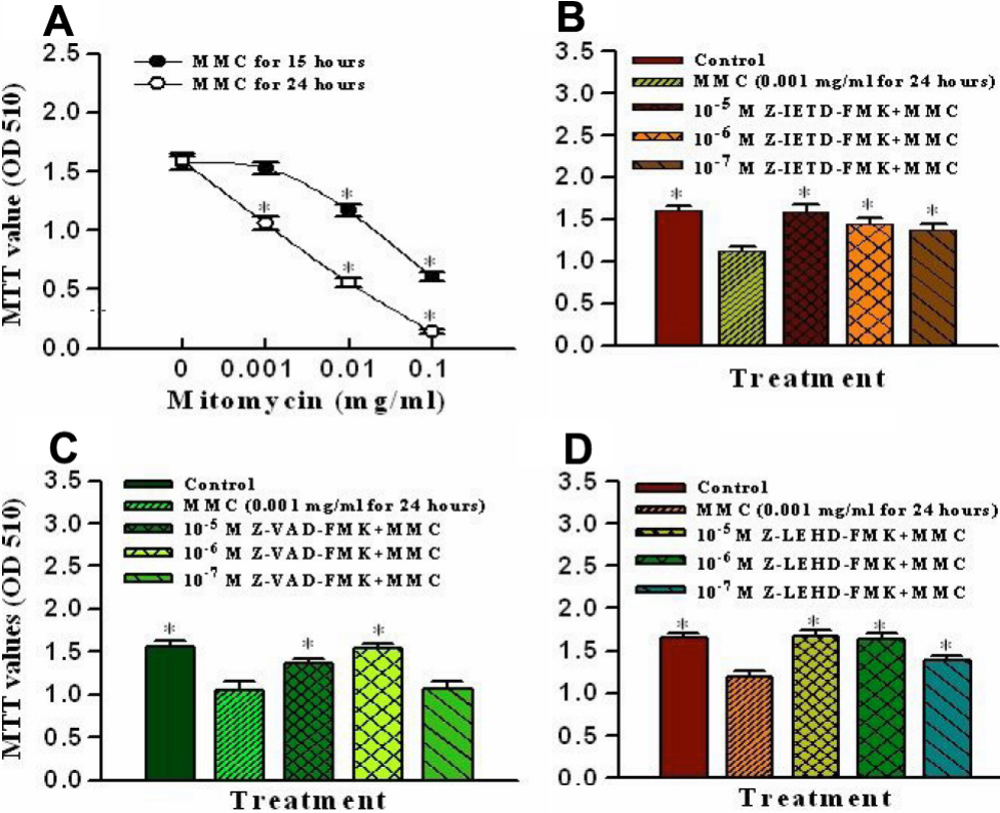
Effects of mitomycin C on cell viability and apoptotic caspase pathways. **A:** Cell viability was measured under exposure to various concentrations of mitomycin C at 15 h and 24 h in cultured porcine corneal endothelial cells. **B**, **C**, and **D**: The mitomycin-induced apoptotic caspase pathways were identified with different caspase inhibitors. The cells were pretreated with a caspase-8 inhibitor (Z-IETD-FMK; **B**), a general caspase inhibitor (Z-VAD-FMK; **C**), or a caspase-9 inhibitor (Z-LEHD-FMK; **D**) for 1 h and then exposed to 0.001 mg/ml mitomycin C for 24 h. Data are presented as means±SD from six replicates and three independent experiments. The asterisk denotes that p<0.05 when comparing the control group in **A** with the MMC only treated group in **B**, **C**, and **D**.

The MTT assay is used as a marker for cell viability. In the present study, MMC produced toxic effects on corneal endothelial cells. After incubation with MMC for 15 h and 24 h, cell viability was significantly decreased in a time-dependent and dose-dependent manner at concentrations ranging from 0.1, 0.01, and 0.001 mg/ml (Figure 1A). To investigate the role of caspase in MMC-induced apoptosis, cells were pretreated with various caspase inhibitors for 1 h then incubated with 0.001 mg/ml MMC for 24 h. After application of caspase inhibitors, the cellular MTT values were significantly increased in comparison with the MMC only group. Figure 1B-D show that MTT values of cells were significantly significantly reduced when comparing the MMC only groups and the control groups. The addition of caspase-8 inhibitor, general caspase inhibitor, and caspase-9 inhibitors (Z-IETD-FMK, Z-VAD-FMK, and Z-LEHD-FMK) at 10^-5^ and 10^-6^ M reversed the MMC-induced cellular damage.

### Assay of mitochondrial membrane potential change after mitomycin C exposure

**Figure 2:**
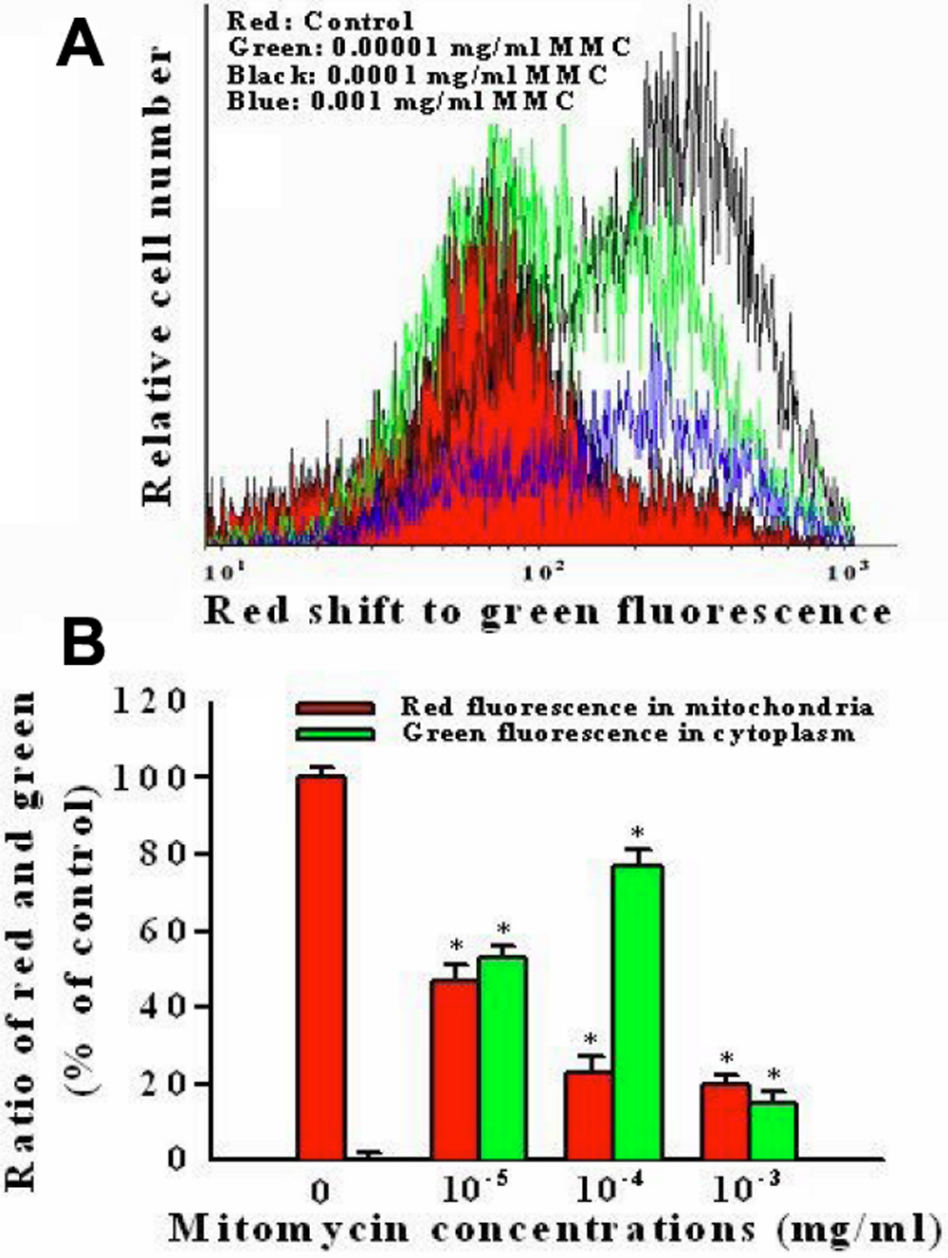
Flow cytomety assay of mitochondrial membrane potential changes with MitoLight dye in cultured porcine corneal endothelial cells under exposure to 0.00001, 0.0001, and 0.001 mg/ml mitomycin C for 24 h. **A:** The curves represent fluorescent changes in the absence of mitomycin C for 24 h (control, red color) or in the presence of 0.00001 (green color), 0.0001 (black color) and 0.001 (blue color) mg/ml mitomycin C. **B**: It is displayed the ratio of red fluorescence in the mitochondria and green fluorescence in the cytoplasm at different concentrations of mitomycin C with histogram. The asterisk denotes that p<0.05 when comparing the red and green fluorescence with control group. Two other independent experiments produced similar results.

Disruption of the mitochondrial transmembrane potential is one of the earliest intracellular changes induced by apoptosis. Following exposure to 0.00001 and 0.0001 mg/ml MMC for 24 h, the fluorescence of MitoLight dye in cells was obviously changed from the mitochondria to the cytoplasm as detected by the shift of the relative fluorescence intensity. Control cells showed red fluorescence, indicating a normal mitochondrial membrane potential, while cells treated with 0.00001, 0.0001 and 0.001 mg/ml MMC showed green fluorescence (Figure 2). The percentage of MitoLight accumulated in the mitochondria was decreased from 100%±3% of the control (red color) to 47%±4% at 0.00001 mg/ml (green color) and 27%±4% at 0.0001 mg/ml (black color). Exposure to 0.001 mg/ml MMC destroyed the mitochondrial membrane potential as shown by the blue color.

**Figure 3:**
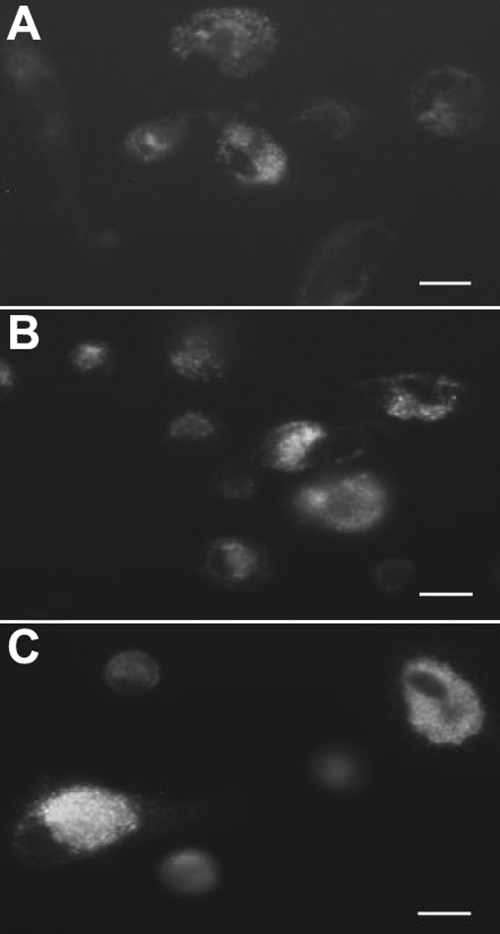
Immunofluorescent staining of cytochrome *c* in cultured corneal endothelial cells. Cells were incubated either in the absence of mitomycin C for 24 h (control, **A**) or in the presence of 0.001 mg/ml (**B**) and 0.01 mg/ml (**C**) mitomycin C for 24 h. The intensity of fluorescence staining with cytochrome *c* was gradually enhanced from the control cells to the apoptotic cells. Apoptotic changes throughout the cytosol were clearly visible following exposure to 0.01 mg/ml mitomycin C for 24 h. The bar in each panel represents 5 μm. Two other independent experiments produced similar results.

Cytochrome *c* released from the mitochondria into the cytoplasm is characteristic of cellular apoptosis. In control cells, the cytochrome *c* protein was found in the mitochondrial membrane (Figure 3A). After exposure to 0.001 and 0.01 mg/ml MMC for 24 h, typical apoptotic cells were recognized that were shrunken in shape and had lost contact with neighboring cells. Exposure to 0.001 mg/ml MMC caused the cytochrome *c* to slightly disperse in the cytoplasm (Figure 3B). Apoptotic changes throughout the cytosol were clearly visible following exposure to 0.01 mg/ml MMC for 24 h (Figure 3C).

### Apoptotic TUNEL staining

**Figure 4:**
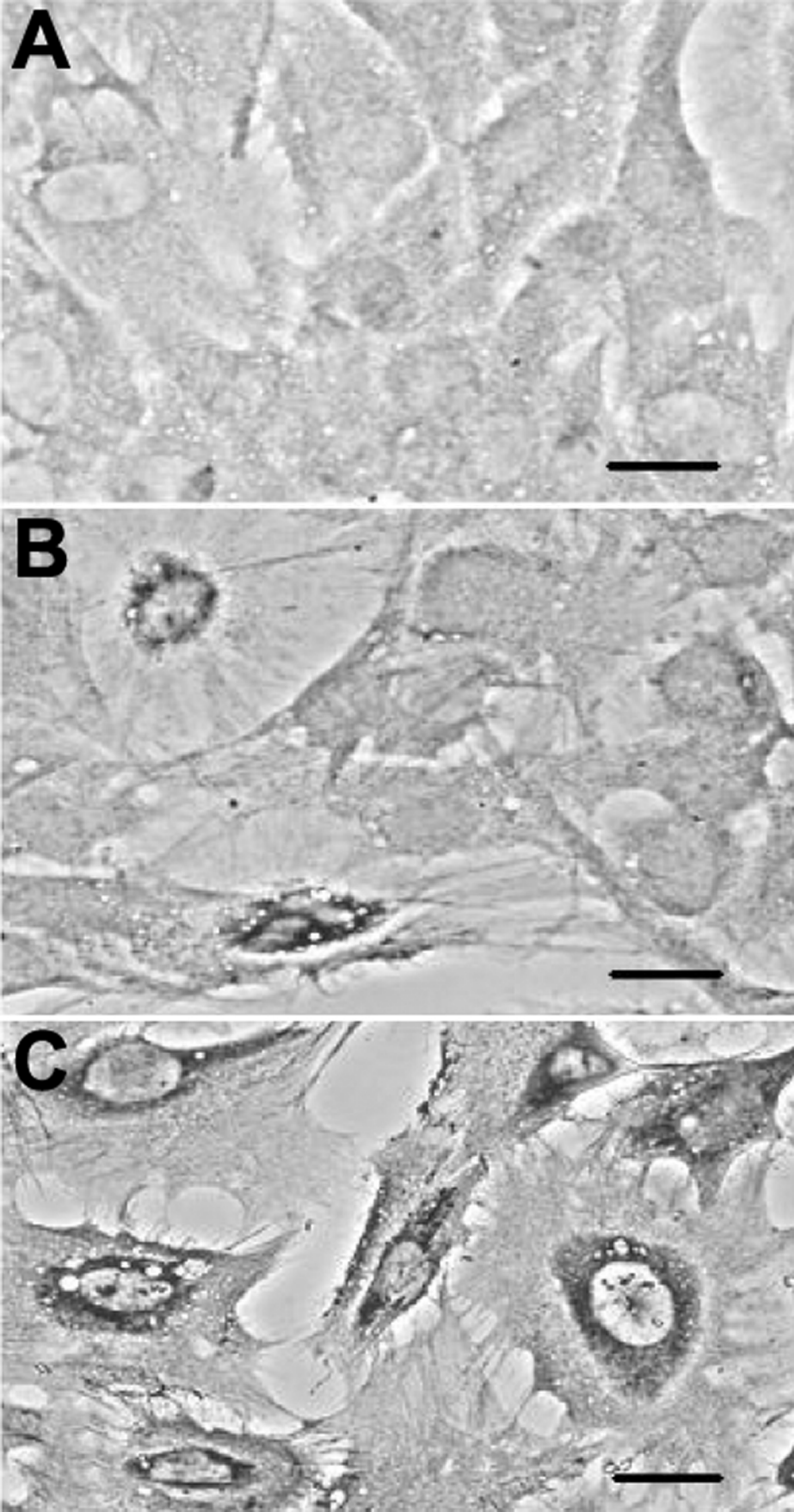
Apoptotic DNA characteristics of corneal endothelial cells visualized with TUNEL staining. Cells were incubated either in the absence of mitomycin C for 15 h and 24 h (control, **A**) or in the presence of 0.001 mg/ml MMC for 24 h (**B**) and 0.01 mg/ml mitomycin-C for 15 h (**C**). The typical positive DNA apoptosis was clearly stained following exposure to mitomycin C. After exposure of cells to 0.001 mg/ml mitomycin C, cellular chromatin condensed and marginalized at the nuclear membrane. After exposure to 0.01 mg/ml mitomycin C, cells were seriously damaged and formed typical apoptotic bodies containing cytosol, condensed chromatin, and organelles. Each bar represents 3 μM. Two other independent experiments produced similar results.

Characterization of apoptosis was performed by TUNEL staining. In comparison to the negative staining observed in control cells (Figure 4A), cells exposed to 0.001 mg/ml MMC for 24 h were seen to contain cellular chromatin condensed and marginalized at the nuclear membrane (Figure 4B). After exposure to 0.01 mg/ml MMC for 15 h, almost all cells were seriously damaged and showed typical apoptotic bodies, which contained cytosol, condensed chromatin, and organelles (Figure 4C).

### Annexin V-FITC/propidium iodide staining in cellular plasma membrane with flow cytometry

**Figure 5:**
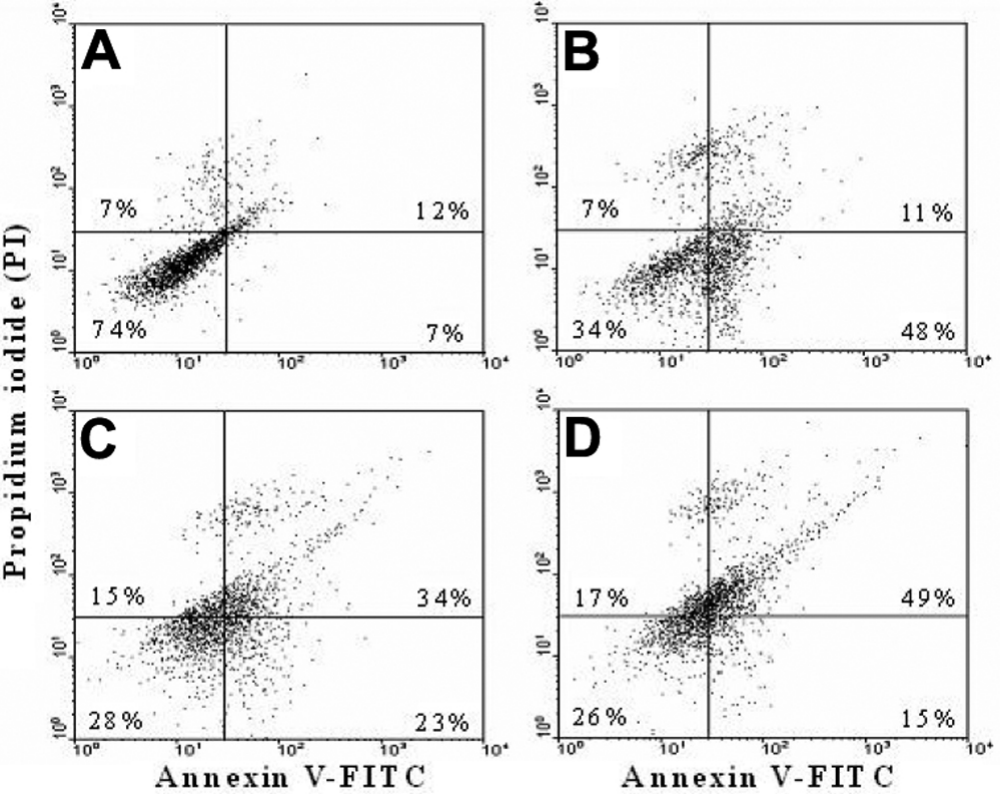
Flow cytometry analysis of plasma membranes with annexin V-FITC/PI double staining. Cells were incubated either in the absence of mitomycin C for 15 h and 24 h (control, **A**) or in the presence of 0.001 mg/ml of mitomycin C for 24 h (**B**) and 0.01 mg/ml mitomycin C for 15 h (**C**) and 24 h (**D**). Undamaged cells were stained with negative annexin V-FITC/PI (bottom left quadrant). After incubation with 0.001 mg/ml mitomycin C for 24 h, a significant number of apoptotic cells were stained with positive annexin V-FITC and negative PI (bottom right quadrant). Following the increasing of mitomycin C to 0.01 mg/ml, advanced apoptotic cells stained by positive annexin V-FITC and PI (upper right quadrant) were significantly augmented from 12%±3% of the control to 34%±3% after 15 h and 49%±4% after 24 h incubation. During advanced apoptosis stages, the cells were no longer viable. Data are presented as means±SD from triplicates and three independent experiments. Two other independent experiments produced similar results.

To identify apoptosis of the plasma membrane, annexin V-FITC/PI double staining in cells was performed by flow cytometry. In the nonapoptotic, viable control cells, the annexin V-FITC staining and PI negative staining were located in the bottom left quadrant of the dots (Figure 5A). After exposure of the cells to 0.001 mg/ml MMC for 24 h, a significant number of cells showed annexin V-FITC positive and PI negative staining, which increased the dot numbers in the bottom right quadrant from 7%±2% of control cells to 48%±3% (Figure 5B). The cells in this stage of apoptosis were still viable. Following the increase of MMC to 0.01 mg/ml, cells in advanced apoptosis stained positive with annexin V-FITC and PI (upper right quadrant) and were significantly augmented from 12%±3% of control cells to 34%±3% after 15 h (Figure 5C) and 49%±4% after 24 h incubation (Figure 5D). The population of cells progressed to advanced apoptosis, indicating that the cells were no longer viable.

### Immunoblot analysis of proteins involved in apoptosis

**Figure 6:**
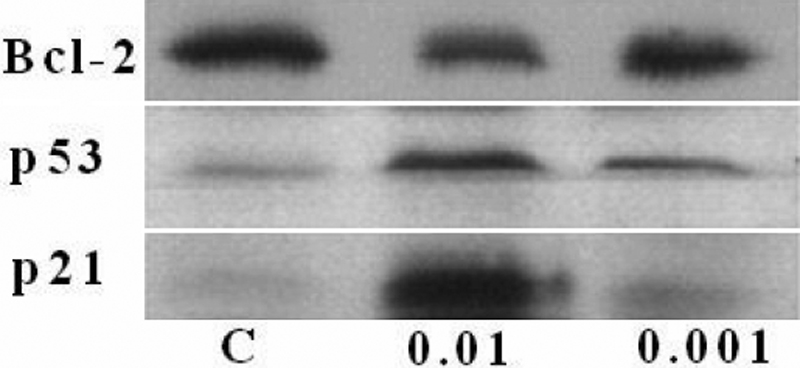
Western blot assay of proteins involved in apoptosis in corneal endothelial cells. Following incubation of cells either in the absence of mitomycin C for 24 h (labeled as C in the image) or in the presence of 0.01 mg/ml (labeled as 0.01 in the image) and 0.001 (labeled as 0.001 in the image) mg/ml mitomycin C for 24 h, cells were subjected to SDS–PAGE electrophoresis and immunoblotting using antisera against Bcl-2, p53, and p21. Densitometric analysis of protein bands showed the optical density values of various proteins with a detailed description in the Results. The respective control values of three proteins were assumed as 100% response. The optical density of the Bcl-2 protein decreased to 64%±4% at 0.01 mg/ml and 86%±5% at 0.001 mg/ml in comparison with the control protein value of 100%±4%. The p53 protein only significantly increased at 0.01 mg/ml to 139%±4% over the control value. The p21 protein increased to 373%±6% at 0.01 mg/ml and 149%±7% at 0.001 mg/ml in comparison with the control value. Two other independent experiments produced similar results.

To investigate the effect of MMC on proteins involved in apoptosis in cultured corneal endothelial cells, we examined the anti-apoptotic protein, Bcl-2, and two apoptotic proteins, p53 and p21, through western blot analysis. The results of three independent experiments demonstrated that MMC significantly decreased the content of Bcl-2 protein and increased the amount of p53 and p21 proteins in a dose-dependent manner (Figure 6). Densitometric analysis of Bcl-2 proteins bands showed that the optical density of proteins in control cells, 0.01 mg/ml MMC-treated cells, and 0.001 mg/ml MMC-treated cells was displayed as 336±8 (100%±4% of the control), 241±7 (64%±4% at 0.01 mg/ml MMC), and 292±9 (86%±5%), respectively. In contrast, the optical density for p53 proteins was 168±6 (control), 253±7 (0.01 mg/ml MMC) and 182±6 (0.001 mg/ml MMC). The protein only significantly increased at 0.01 mg/ml (139%±4% of the control). In the presence of 0.01 and 0.001 mg/ml MMC for 24 h, optical density of p21 proteins were 111±7 (control), 415±9 (0.01 mg/ml MMC), and 166±6 (0.001 mg/ml MMC), which corresponded to an increase of 373%±6% for 0.01 mg/ml and 149%±7% for 0.001 mg/ml over control values.

## Discussion

In the present study, the mechanism of MMC-induced apoptosis in corneal endothelial cells was investigated using cultured porcine cells. This work presents the findings that MMC treatment not only decreases cell viability but also induces apoptosis. The effects included turnover of membrane phosphatidylserine, as identified by annexin V-FITC, DNA degradation, as characterized with TUNEL staining, changes in membrane potential, as characterized by the release of cytochrome *c* from mitochondria, and upregulation of p53 and p21 proteins, caspase-8 and caspase-9.

Application of MMC for the treatment of various ocular diseases has become more and more popular. However, it is not easy to detect obvious damage to corneal endothelial cells unless the side effects of MMC are severe enough to interfere with normal cellular physiologic function. A recent study has reported that a significant loss of corneal endothelial cells was observed after trabeculectomy with adjunctive MMC in glaucoma patients [7]. Evidence has also shown that the corneal endothelial cell count in patients was significantly decreased [16] and that permanent corneal edema was induced after photorefractive keratectomy by topical use of MMC [17]. Some earlier studies also found that the aqueous concentration of MMC linearly increased with increases in the drug’s exposure time and concentration [18] and that a dose-dependent increase in corneal thickness, decrease in corneal clarity, and increase in corneal endothelial apoptosis occurred after intraoperative application of MMC in an animal study [8].

Many side effects of MMC often occur only after a period of clinical use. For example, dying corneal endothelial cells displayed cell shrinkage and chromatin condensation three weeks after photorefractive keratectomy [19], suggesting a high possibility that cells underwent an apoptosis process induced by MMC. Previous studies have reported that MMC-induced apoptosis in rabbit corneal keratocytes is mediated through the caspase-8 and caspase-9 pathways and the release of cytochrome *c* protein from the mitochondria [20,21]. In other ocular tissues, such as human lens epithelial cells, MMC also induces the upregulation of Bax, p53, and caspase-3 [22], and in human Tenon's capsule fibroblasts, it also increases caspase-3, caspase-9, p53, Fas, FasL, and Bad as well as inducing the release of cytochrome *c* and changes in the mitochondrial membrane potential [23]. However, the mechanism of apoptosis induction by MMC in any tissue, including corneal endothelial cells, is poorly understood at present.

Based on the dose response of MMC in corneal endothelial cells, we found that disruption of the mitochondrial transmembrane potential was the first apoptotic characteristic detected. The mitochondrial depolarization occurs without necessarily going into the cell death pathway. The down-regulation of Bcl-2 levels, the anti-apoptotic protein, may accelerate damage to the mitochondria. The mitochondria then releases the pro-apoptotic factors such as cytochrome *c* from the inner mitochondrial membrane into the cytosol, which then activates the caspase-9 cascade. Thus, mitochondrial depolarization is an early event marker to more severe apoptotic events.

Our data illustrated that MMC induced cellular DNA fragmentation and then triggered p53 and p21 expression. MMC damages DNA by cross-linking bases in the same or adjacent strands of DNA, which eventually induces a powerful stimulus such as p53 for apoptosis [24,25]. Then, p53 directly activates the expression of a large panel of genes such as p21, which plays a major role in mediating p53-dependent cell cycle G_1_ arrest [26,27].

Utilization of annexin V-FITC to identify apoptosis in the plasma membrane indicated that early apoptotic cells were clearly stained by annexin V-FITC after 24 h of incubation in 0.001 mg/ml MMC. Following exposure to 0.01 mg/ml MMC for 24 h, about 20% of the cells were still stained by PI and annexin V-FITC. Comparing the apoptotic dose response for the mitochondria and DNA with that for the plasma membrane, the cellular plasma membrane appears to be the most resistant to MMC-induced damage.

The apoptotic caspases are activated by two signal complexes either in response to the ligation of cell surface death receptors (called extrinsic apoptosis pathways) or in response to signals originating from the mitochondria (called intrinsic apoptosis pathways) [23,28]. Evidence has shown that treatment with MMC increased the expression of Fas and FasL, which belong to the death receptor pathway [23]. Once these receptors are activated, caspase-8 is activated for the execution of apoptosis [29]. Our data indicated that both caspase-8 and caspase-9 inhibitors reversed the MMC-induced damage, suggesting that MMC-induced apoptosis in corneal endothelial cells may occur through both intrinsic mitochondrial-mediated and extrinsic death receptor-mediated caspase activation. However, the MMC-induced extrinsic apoptosis pathway in particular needs to be explored further in corneal endothelial cells.

In summary, this study has demonstrated that MMC not only results in cytotoxicity in a dose-dependent and time-dependent manner but also induces apoptosis in corneal endothelial cells through activation of intrinsic mitochondrial and extrinsic caspase-8 apoptotic pathways. The appearance of apoptotic characteristics in corneal endothelial cells may extend the chronic toxicity of MMC to cells. Thus, the use of MMC may need to be carefully monitored for adverse changes in corneal endothelial cell viability.
